# Peptidomic profiles of post myocardial infarction rats affinity depleted plasma using matrix-assisted laser desorption/ionization time of flight (MALDI-ToF) mass spectrometry

**DOI:** 10.1186/2001-1326-1-11

**Published:** 2012-06-15

**Authors:** Bing Hui Wang, Simone Reisman, Mark Bailey, Andrew Kompa, Mustafa Ayhan, Henry Krum, Gregory Rice

**Affiliations:** 1 Centre of Cardiovascular Research and Education in Therapeutics, Department of Epidemiology & Preventive Medicine, Monash University, Melbourne, VIC, 3004, Australia; 2 Centre for Clinical Research, University of Queensland, Bld 71/918, Royal Brisbane and Women’s Hospital, Herston, QLD, 4032, Australia; 3 Department of Medicine, The University of Melbourne, St Vincent’s Hospital, 41 Victoria Parade, Fitzroy, VIC, 3065, Australia

**Keywords:** Myocardial infarction, Peptidomic profiling, Mass spectrometry, Biomarkers, Heart failure

## Abstract

**Background:**

Despite major advances in drug development, effective cardiovascular therapies and suitable cardiovascular biomarkers remain limited. The aim of this study was to leverage mass spectrometry (MS) based peptide profiling strategies to identify changes that occur in peptidomic profiles of rat plasma following coronary artery ligation generated myocardial infarction (MI).

**Methods:**

One week after MI, rats were randomized to receive either an ACE inhibitor (ramipril, Ram-1 mg/kg/day), or vehicle (Veh) for 12 weeks. Echocardiography and hemodynamic measurements were made before sacrifice and plasma collection. High abundance proteins were depleted with affinity capture before MS profiling. Differentially expressed peptide ions were identified using proprietary software (ClinProtTools).

**Results:**

MI increased heart/body weight (18%), lung/body weight (56%), and left ventricular (LV) end diastolic pressure (LVEDP, 247%); and significantly reduced percentage fractional shortening (FS, 75%) and rate of pressure rise in the LV (dP/dtmax, 20%). Ram treatment significantly attenuated the changes in LVEDP (61%) and FS (27%). Analysis of MALDI-ToF generated mass spectra demonstrated that peptide ions 1271, 1878, 1955, 2041 and 2254 m/z were consistently decreased by Ram treatment (p < 0.001) and thus may be associated with the agent’s therapeutic effects. Among peptides that were significantly changed, synapsin-2, adenomatous polyposis coli protein and transcription factor jun-D were identified as significantly reduced by Ram treatment.

**Conclusions:**

This approach allows us to screen for potential biomarkers in a window of the blood proteome that previously has been difficult to access. The data obtained from such an approach may potentially useful in prognosis, diagnosis, and monitoring of treatment response.

## Background

Despite the advancesment in the development of effective therapies for cardiovascular diseases (CVDs), their socioeconomic and human costs continue to escalate throughout the world, with an aging population. The number of effective cardiovascular therapies and viable therapeutic targets remains surprisingly limited. The number of clinically useful cardiovascular biomarkers is even fewer [1]. As most CVDs directly impact expression and function of circulating proteins and peptides, the likelihood of identifying CVD-associated changes within this system is significant.

Considerable insight has emerged over the past few decades with regard to cellular and molecular derangements within the myocardium. Analysis of gene expression, while a powerful tool, often reveals a poor correlation with the quantity of corresponding functional protein. This is probably related to the divergent time course of gene expression in relation to protein expression, regulation and downstream protein production as well as the involvement of other factors. Given the rapid advances in proteomic profiling over the last few years, recent editorials have called for direct and large-scale analysis of proteins within the failing myocardium where significant information may be gained from proteomic approaches [2-4].

With the wide array of cellular dysfunction observed in failing myocardium in the whole animal setting as well as derangements in individual cell types, it is expected that there will be considerable differences in peptide profiles between normal and dysfunctional myocardium. Indeed, peptide profiling approaches (peptidomics) to identify disease- and treatment-associated changes are delivering novel insights [5,6].

In the current study, mass spectrometry-based peptide profiling strategies were utilized to identify and characterize changes in the peptidomic profiles of blood associated with myocardial infarction (MI) and heart failure (HF). We employed an experimental animal model of the disease aiming to identify characteristic peptidomic profiles that may be useful for disease prognosis, diagnosis and/or monitoring of treatment response. The approach we have developed allows screening for biomarkers of MI and HF in a window of the blood proteome that previously has been difficult to access with conventional proteomic strategies. The strength of this approach is that we also have the opportunity to simultaneously identify peptide peaks detected at differential levels.

Considering the natural variability expected between human samples due to back gound therapies, to adequately test hypotheses relating to changes in plasma peptidomic profiles during disease progress and in response to specific treatment regimens, a proven animal model of disease is essential. Animal models of MI involving ligation of the left anterior descending (LAD) coronary artery have been widely used for studies of functional and therapeutic effects in left ventricle remodeling and HF. Our laboratory has established and routinely performed studies with this model of post-MI HF in rats [7-10]. We have previously demonstrated beneficial effects of p38-MAPK inhibition on left ventricular remodeling and cardiac function in this HF model [7,8]. We, therefore, have used this model to define temporal changes in peptidomic profiles during disease progression.

Current therapeutics, such as angiotensin-converting enzyme (ACE) inhibitors, attenuate left ventricle remodeling in both animal models and in patients with post-MI and HF [8,11-14]. ACE inhibitors can prevent cardiac myocyte hypertrophy and cardiac fibrosis, reduce ventricular dilation and cardiac dysfunction and prolong survival after MI [7,8,11-13]. Therefore, treatment of post-MI rats with the ACE inhibitor, ramipril (Ram), provides an ideal model for investigating potential changes in peptide profiles that may be indicative of therapeutic benefits and/or drug induced responses. The primary hypotheses to be tested in this study were: that post-MI rats which develop HF display altered plasma peptide profiles; and that therapy with the ACE inhibitor ramipril (Ram) may reverse/affect such changes.

## Method and materials

The authors of this manuscript have certified that they comply with the Principles of Ethical Publishing in the International Journal of Cardiology: Shewan LG and Coats AJ. Ethics in the authorship and publishing of scientific articles. Int J Cardiol 2010;144:1–2.

### Study design

#### Myocardial infarction experimental protocol and blood sample collection

Female Sprague Dawley rats (180–220 g) underwent coronary artery ligation under anesthesia as previously described [8]. One week after myocardial infarction (MI) rats were randomized to receive either an angiotensin converting enzyme inhibitor (Ram – 1 mg/kg, MI + Ram), or vehicle (0.5% methylcellulose, MI + Veh) by gavage, daily for 12 weeks. The control group consisted of sham-operated animals (Sham) without coronary artery ligation receiving vehicle for 12 weeks. At the end of the treatment period, echocardiographic images were obtained and hemodynamic measurements by cardiac catheterization were recorded prior to animal sacrifice and tissue collection. All experiments were performed in accordance with the Australian National Health and Medical Research Council, Code of Practice for the Care and Use of Animals for Scientific Purposes (7^th^ edition, 2004), with approval of the AMREP Animals Ethics Committee at the Alfred Hospital.

#### Echocardiography and cardiac catheterization

Echocardiography was performed in lightly anaesthetized animals (ketamine 40 mg/kg, xylazine 5 mg/kg, i.p.) after 12 weeks treatment using a HP Sonos 5500 with a 12 MHz probe (Agilent Technologies, Palo Alto, CA, USA). Two-dimensional and m-mode images at the mid-papillary muscle level were used to obtain LV internal diameters in systole (LVIDs) and diastole (LVIDd) which were measured offline and % LV fractional shortening determined [8].

Following final echocardiography, animals were sedated with pentobarbitone (50 mg/kg, ip) and hemodynamic measurements of central aortic pressure (CAP), systolic (SBP) and diastolic (DBP) blood pressure, heart rate (HR), LV end-diastolic pressure (LVEDP), and the maximal (dP/dt_*max*_) and minimal (dP/dt_*min*_) first derivative of left ventricular pressure were obtained using a pressure transducer (UFI, model 1050, Morro Bay, CA, USA) connected to a MacLab system (ADInstruments, Castle Hill, NSW, Australia)[8]. On completion of these measurements the heart and lungs were weighed, blood was collected in K+/EDTA tubes and centrifuged at 4°C, 3000RPM for 15 min. Plasma aliquots were frozen and stored at −80°C for proteomic studies.

#### Statistical analysis for functional data

Integrated Peak Area (IPA) is expressed as mean ± SEM, with 8 animals in each group. Differences between groups were compared using an ANOVA, p values < 0.05 were considered statistically significant.

#### Removal of high abundance proteins

Prior to analysis, high abundance proteins were removed using Affi-Gel Blue and Protein A (Bio-Rad) [15]. Plasma samples (60μL, 60–80 mg protein/mL) were diluted in 180μL 20 mM phosphate buffer (pH7.0). Diluted plasma was then incubated with 400μL Affi-Gel Blue (Bio-Rad Laboratories, Hercules, CA) and 60μL Affi-Gel Protein A (Bio-Rad) for 30 min at room temperature in a micro-spin column (Bio-Rad). Samples were mixed every 5 min. Following incubation the flow-through (under gravity) was collected (~200μL) and stored at −80°C for protein determination and analysis.

#### Sample preparation with solid phase extraction

High-abundance protein depleted plasma supernatants (100μL, 250–300μg protein) were cleaned up using solid phase extraction (SPE). The SPE plates were Oasis HLB μelution plates (Waters Corporation, Milford, MA) containing a water-wettable reversed phase polymer (poly(divinylbenzene-co-N-vinylpyrrolidone)) also referred to as hydrophilic-lipophilic-balanced (HLB), as previously reported by other groups. [16,17] Briefly, the SPE plate was conditioned (methanol, 300μL); followed by 300μL, 75%:0.1%, acetonitrile:formic acid (ACN:FA), then equilibrated (ACN:FA, 2%:0.1%, 300μL). Depleted plasma (100μL) was acidified with FA (100%, 1μL) and applied to the wells. Wells were washed (6 times, ACN:FA, 2%:0.1%, 300μL) and protein was eluted (ACN:FA, 60%:0.1%, 50μL) into a new microtitre plate (Greiner, Stonehouse, UK).

#### Peptide profiling mass spectrometry

Samples eluted from SPE plates were analyzed using MALDI-ToF mass spectrometry (Autoflex II, ToF/ToF, Bruker Daltonics, Bremen, Germany) in linear-positive mode equipped with a 337 nm nitrogen laser and 2 GHz digitizer. Each SPE eluted sample was applied to the targetplate in quadruplicate, assuring 4 independent measurements of each sample. Mass spectra were collected under 20 kV of ion acceleration with the ion deflection at mass ≤ 1000 m/z and time lag focusing at 120 nanoseconds. The mass range was calibrated using a calibration mixture [angiotensin I (1296.684), angiotensin II (1046.541), substance P (1347.735), bombesin (1619.822), ACTH clip1-17 (2093.086), ACTH clip18–39 (2465.198), somatostatin 28 (3147.471), insulin (5734.52), ubiquitin (8565.76)]. Spectral data was collected in automatic acquisition mode over an *m/z* range of 1000–10,000 m/z using the “AutoXecute” function (FlexControl ™, version 2.2). Thirty-three sets of 30 shots (at 25 Hz frequency) were collected to give a comprehensive coverage for each sample spot. Each spectrum represents the average of 990 laser shot at a fixed laser energy output.

#### Data analysis of peptidomic profiles

Raw spectral files were submitted to proprietary software (ClinProtTools, v2.0, Bruker Biosciences) to identify peptide ions detected at differentially expressed levels. ClinProTools generates pattern recognition models for classification and prediction and allows graphical visualization of the processed spectral profiles and generation of statistical classification models. The program prepares the spectral data using a series of standard processing steps; baseline subtraction, normalization, recalibration, calculation of Total Average Spectrum (TAS), calculation of peak area on TAS, calculation of area of each peak and normalization of peak areas. Peak statistics are generated from the processed data. The peak comparison output represents (i) peak intensity and (ii) 2D peak distribution standard deviation envelopes (95% confidence interval). Spectral data presented represent mean peak intensity and a threshold signal-to-noise ratio of 30 (based upon the averaged spectra) was used for peptide peak definition.

#### Peptide identification – LC MS/MS

The peptides eluted from SPE were pooled and dried in a vacuum centrifuge. These were resuspended in 20μL of 2% ACN/0.1% FA and 8μL was applied to a C18 RP ProteoCol trap cartridge (10 mm × 150 μm, 300 Å pore size, SGE Analytical Science, Victoria, Australia) using an Agilent 1100 capillary HPLC (Agilent, Foster City, CA) coupled to a 3D ion-trap mass spectrometer (HCT Ultra, Bruker Daltonics). The column was equilibrated with Buffer A (Buffer A: 0.1%FA, 10mins, Flow rate 4μL/min) prior to gradient separation (2–60%) using Buffer B (90%ACN/0.1%FA, 4μL/min). Buffer B was then increased over 5mins from 60–100% and was held for 5 min at 100%B before reducing to initial conditions (100% Buffer A, 5mins) and holding for a further 5 min. The ion trap was used in standard scan mode. Total ion current chromatograms and MS/MS mass spectra were acquired using Esquire control interface (Bruker Daltonics). Tandem mass spectra were extracted using DataAnalysis (Bruker Daltonics, Version 3.3). Biotools software (Bruker, Version 3.1) and the Mascot search engine (Matrix Science, London, UK; version 2.2) were used to interrogate the SwissProt database (Release 57.12). Search parameters were: Taxonomy: Rat; parent ion mass tolerance: 2.4 Da; fragment ion tolerance: 1.2 Da; Missing Cleavages: 0; Enzyme: none; Variable Modifications: Oxidation (M). The Peaks software package (Bioinformatics Solutions, Waterloo, Ontario, Canada) was also used to analyze MS/MS data. The Bruker analysis.yep file was imported directly into Peaks. Mass spectral data was preprocessed with filter quality was set at >0.65, charge options 1–3, and with other options appropriate for the Bruker Ion Trap. The processed data were used to search the SwissProt database (Release 57.9). Search parameters were: Taxonomy: Rat; parent ion mass tolerance: 2.4 Da; fragment ion tolerance: 0.8 Da; Max missed cleavages: 100; Enzyme: none; Variable Modifications: Oxidation (M); Max variable PTM per peptide: 3. Peptide identified via Peaks and Mascot search engines masses were match with masses identified from the MALDI-ToF profiles via ClinProTools with a signal to noise 15:1.

## Results

### Organ weights and infarct size

There were no significant increases in heart weight to body weight ratio (HW/BW) amongst the groups, however a strong trend toward increased hypertrophy (18%) was observed in MI + vehicle animals compared to sham animals. Ram treatment attenuated this increase of hypertrophy (Table 1). Lung weight to body weight ratio (LW/BW) was increased significantly by 56% in MI vehicle animals, indicative of the pulmonary congestion that accompanies heart failure. Ram attenuated this increase in LW/BW (Table 1). Infarct sizes were similar between the MI groups (Table 1).

**Table 1:** Tissue, Body weights and Infarct Size

	**Sham (n = 8)**	**MI + Veh (n = 8)**	**MI + Ram(n = 8)**
**Body weight (g)**	292 ± 5	291 ± 5	307 ± 10
**Heart weight (g)**	0.92 ± 0.02	1.09 ± 0.11	1.03 ± 0.06
**Lung weight (g)**	1.06 ± 0.02	1.65 ± 0.29 *	1.22 ± 0.12
**HW/BW (mg/g)**	3.16 ± 0.06	3.75 ± 0.32	3.34 ± 0.10
**LW/BW (mg/g)**	3.63 ± 0.10	5.66 ± 0.94 *	3.98 ± 0.41
**Infarct Size (%)**	---	50.7 ± 3.8	50.2 ± 1.9

Data are expressed as mean ± SEM, * P < 0.05 compared to sham animals, n = 8 in each group. HW/BW: heart weight/body weight; LW/BW: lung weight/body weight.

### Echocardiography and hemodynamic parameters

Percentage fractional shortening (FS) was reduced by 75% in MI + Veh animals; treatment with Ram significantly attenuated this reduction by 26% (Table 2). LVIDs and LVIDd were increased in MI + Veh animals by 107% and 43% respectively; treatment with Ram significantly attenuated the increase in LVIDs and LVIDd by 26% and 17% respectively (Table 2).

**Table 2:** Echocardiography and Hemodynamic assessment

	**Sham (n = 8)**	**MI + Veh (n = 8)**	**MI + Ram (n = 8)**
*Echocardiography data*			
**LVIDs (cm)**	0.43 ± 0.02	0.89 ± 0.02 ***	0.77 ± 0.02 ^###^
**LVIDd (cm)**	0.69 ± 0.01	0.99 ± 0.01 ***	0.94 ± 0.02 ^#^
**%FS**	38.6 ± 2.1	9.8 ± 1.3 ***	17.2 ± 2.0 ^#^
*Hemodynamic data*			
**CAP (mmHg)**	127.6 ± 1.7	117.1 ± 5.1	104.3 ± 3.4
**SBP (mmHg)**	146.9 ± 4.0	132.5 ± 4.4	120.1 ± 2.5 ^#^
**DBP (mmHg)**	106.8 ± 3.3	102.0 ± 6.8	88.9 ± 4.4
**HR (beats/min)**	348 ± 7	355 ± 29	352 ± 16
**LVEDP (mmHg)**	2.8 ± 0.7	9.1 ± 1.1 ***	5.5 ± 0.8 ^#^
**dP/dt*max*(mmHg/s)**	5898 ± 165	4819 ± 274 *	5156 ± 115
**dP/dt*min*(mmHg/s)**	−5851 ± 161	−4637 ± 329 **	−5021 ± 159

LVIDs – left ventricular internal diameter in systole; LVIDd – left ventricular internal diameter in diastole; %FS – percentage fractional shortening; CAP – central aortic pressure; SBP – systolic blood pressure; DBP – diastolic blood pressure; HR – heart rate; LVEDP – left ventricular end diastolic blood pressure; dP/dt*max* – rate of left ventricular pressure rise; dP/dt*min* – rate of left ventricular pressure fall.

Data are expressed as mean ± SEM; * P < 0.05, ** P < 0.01, *** P < 0.001 compared to sham animals; ^#^ P < 0.05, ^###^ P < 0.001 compared to MI + Veh animals.

Hemodynamic analyses revealed no significant differences in central aortic, systolic and diastolic blood pressure between the groups. MI + Veh animals had a lower blood pressure compared to the sham group but this was not significant. Ram treatment significantly reduced systolic blood pressure further by 9.4% compared to MI + Veh animals (P < 0.05; Table 2).

LVEDP was increased by 225% in MI + Veh animals compared to the sham group, Ram treatment reduced this increase by 57% (P < 0.05; Table 2). dP/dt*max* and dP/dt*min* were reduced in MI + Veh animals by 18% and 21% respectively compared to the sham group (P < 0.05; Table 2), Ram treatment showed slight improvement of these measures but did not reach significance (Table 2).

Thus, 3 months post-MI, the MI + Veh group had developed HF while treatment with Ram significantly improved cardiac function providing an ideal HF animal model for peptidomic profiling.

#### Protein depletion

Total protein was determined before and after Affi-Gel Blue/Protein A depletion of whole plasma. Values averaged 44.69 ± 8.36 mg/ml protein (before depletion) and 0.94 ± 0.15 mg/ml protein (after depletion). This represents an average depletion of approximately 83% of total protein per sample.

#### Peptide profile analysis

**Figure 1:**
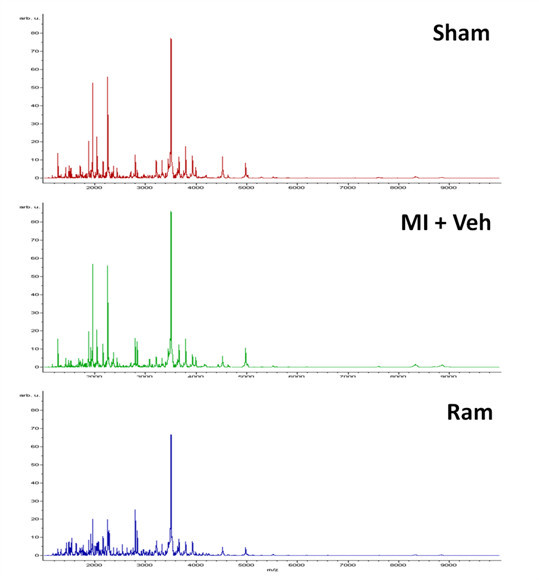
**Plasma Peptide Profile.** Average MALDI TOF mass spectra (over the range 1000 to 10,000 m/z) of plasma peptides eluted from SPE. Samples were AGB depleted plasma from experimental groups sham (n = 8), MI + Veh (n = 8) and MI + Ram (n = 8). Data are presented as relative peak intensity vs. m/z.

The primary hypothesis to be tested in this study was that post-MI rats that developed HF display altered plasma peptide profiles. Plasma samples (24 total, n = 8 for controls, MI + Veh and MI + Ram) were subjected to Affi-Gel blue and Protein A for removal of high abundance proteins followed by SPE. Peptides eluted from SPE were analyzed by mass spectrometry, a mass spectrum was collected for each sample and a protein profile over the mass range 1000–10,000 m/z was created from the averaged spectra of each group (Figure 1, data is presented as relative peak intensity versus m/z). When control (sham), disease state (MI + Veh) and treatment (Ram) were compared predominant peptide peaks were observed in the mass range 1000–5000 m/z.

#### Statistical analyses

**Figure 2:**
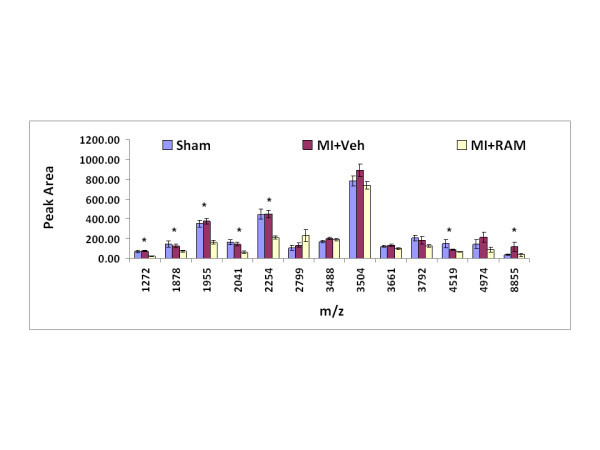
**Average integrated peak area ± SE for 13 ion peaks from 3 experimental groups; Sham (n = 8), MI + Veh (n = 8) and Ram (n = 8) is displayed.** * indicates statistically significant IPA between all groups (ANOVA, p < 0.05).

Peptide profiles were generated for plasma samples obtained from Sham, MI animals (MI + Veh) and MI animals with treatment of Ram (MI + Ram) and mass spectra from the MALDI-ToF were analyzed using ClinProTools. The average integrated peak area ± SE for 13 selected ion peaks are presented in Figure 2. This figure indicates some of the peptides that were detected at differential levels for rats within the three groups, Sham (n = 8), MI + Veh (n = 8) and MI + Ram (n = 8). Differences between these ions were compared based on peak area, with peaks at 1271, 1878, 1955, 2041, 2254, 4519 and 8854 m/z being statistically significant between groups (ANOVA, p values < 0.05). In the MI + Ram group, peptides ion measurements at 1271, 1878, 1955, 2041, 2254, 4519 and 8854 m/z consistently decreased with Ram treatment when compared to samples isolated from MI + Veh rats (p <0.05). The peptide at 2798 m/z was elevated in plasma from MI + Ram rats compared to MI + Veh rats but not statistically significant between groups. Peptides at 1271, 1878, 1955, 2041, 2254, 4519 and 8854 m/z are likely to be associated with therapeutic effects of Ram and the peptide at 2798 m/z may be associated with drug-stimulated effects (Figure 2).

### Peptide identifications

In order to determine the identity of the peptides detected at differential levels, LC-MS/MS ion trap mass spectrometry was used (Bruker Daltonics, Bremen, Germany). Peptide identifications were generated using both Mascot and Peaks software packages. The differentiated expressed peptides identified include Synapsin-2, Adenomatous polyposis coli protein, Transcription factor jun-D, CaiB/baiF CoA-transferase family protein C7orf10 homolog, Estradiol 17-beta-dehydrogenase 1, Contrapsin-like protease inhibitor 1 precursor, Regulator of differentiation 1 and Pyrin (Marenostrin) (Tables 3 and 4).

**Table 3:** Peptide sequences identified from rat plasma

**MALDI M/Z**	**Peptide Sequence**	**Mass (Calc)**	**Δ MALDI MZ - Monoisotopic Mass (Calc)**
1955.00	**QVNASSSSNSLAEPQAPQAA**	1955.9184	−0.92
2041.00	**IVSSLHQAAAAAACLSRQASS**	2041.0374	−0.04
2041.00	**ALSGLAAGASSVAGAAGAPGGGGFAP**	2041.023	−0.02
2799.00	**M[1]HITGPEDGDPVRPGVAM[1]TDLATGLFA**	2799.3203	−0.32
3488.00	**PFHEVYCASKFALEGLCESLAILLPLFGVHVS**	3488.7876	−0.79
3504.00	**LSQPEDQAEINTGSALFIDKEQPILSEFQEK**	3503.7307	0.27
3504.00	**PTGDGQPSLEPPMAAAFGAPGIMSSPYAGAAGFAPAI**	3503.6377	0.36
3661.00	**LHRAETMTASELLGIPPGVKEKLHLLYQKSKSA**	3661.0025	0.00

**Table 4:** The peptides identified from both Mascot and Peaks

**Mass (Calc)**	**Accession Number**	**Protein**	**Protein Name**	**MASCOT Ion score**	**PEAKS Ion Score (%)**
1955.9184	Q63537	SYN2_RAT	Synapsin-2	33	
2041.0374	P70478	APC_RAT	Adenomatous polyposis coli protein		81
2041.023	P52909	JUND_RAT	Transcription factor jun-D		98.6
2799.3203	Q68FU4	CG010_RAT	CaiB/baiF CoA-transferase family protein C7orf10 homolog		43.5
3488.7876	P51657	DHB1_RAT	Estradiol 17-beta-dehydrogenase 1	34	
3503.7307	P05545	CPI1_RAT	Contrapsin-like protease inhibitor 1 precursor	51	
3503.6377	Q9Z118	ROD1_RAT	Regulator of differentiation 1	57	
3661.0025	Q9JJ25	MEFV_RAT	Pyrin (Marenostrin)	34	

## Discussion

Utilizing novel mass spectrometry-based peptide profiling strategies developed in our laboratory, we were able to identify and characterize changes in the peptidomic profiles of blood associated with MI and HF in an experimental animal model of the disease. This approach allows us to screen for potential biomarkers of MI and HF in a window of the blood proteome that previously has been difficult to access due to high abundant proteins masking the identification of lower concentrations of small peptides. In addition, this approach gives us the ability to identify peptide peaks of interest. The present study demonstrated that post-MI rats, which developed HF, display altered plasma peptide profiles when compared to controls. Furthermore, therapy with ACE inhibitor, Ram, reverses some changes but also induces additional alteration, presumably related to its pharmacological effects (Figure 2).

Tandem mass spectrometry allowed us to identify peptides associated with the development of HF in post-MI rat. These include synapsin-2, adenomatous polyposis coli protein, transcription factor jun-D, CaiB/baiF CoA-transferase family protein C7orf10 homolog, estradiol 17-beta-dehydrogenase 1, contrapsin-like protease inhibitor 1 precursor, regulator of differentiation 1 and pyrin (marenostrin) (Table 4). Although identified for the first time from the post-MI plasma as potential biomarkers for post-MI HF, many of them have known functional roles in the cardiovascular system. For example, adenomatous polyposis coli protein has been reported to play an important role in human cardiac development and disease. [18] Transcription factor jun-D is regulated by redox and hypoxia in cardiac myocytes. [19,20] Furthermore, both adenomatous polyposis coli protein and transcription factor jun-D were significantly reduced by the ACE inhibitor, Ram, in post-MI rat plasma (Figure 2, Tables 3 and 4), indicating that the strategy employed in this study is capable of detecting therapeutic response-related peptide changes. It is worth noting that well characterized biomarkers for HF such as arterial natriuretic peptide (ANP) and brain natriuretic peptide (BNP) were not in the list of the potential biomarkers identified in this study. This could be because these peptides were not isolated using our methods, that they were below the limits of detection for our method or that they do not ionize or fragment with sufficient efficiency in the mass spectrometer.

There have been considerable efforts in determining the protein profile changes in both HF patients and the post-MI animal model. Several these studies have identified plasma proteins that are related to cardiovascular functions, including the HUPO plasma proteome project. [21] This project identified some 3020 proteins from healthy subjects with a subset of these proteins having cardiovascular related functions. [21] These proteins include markers of inflammation and/or cardiovascular disease, vascular and coagulation, signaling, growth and differentiation, cytoskeletal, transcription factors, channels/receptors and heart failure and remodeling providing a base line database for the mining of potential biomarkers in the disease stage. This proteomic approaches have also allowed the discovery of previous unknown proteins involved in cardiovascular diseases. The proteomic analysis of plasma from patients with HF or acute coronary syndrome and other cardiovascular diseases [22-27] identified targets suitable for use as biomarkers in inflammation, oxidation stress, extracellular-matrix remodeling, neurohormonal activation, myocyte injury and stress. Proteomic studies utilizing plasma from acute MI patients have identified cardiac troponin I and α1-chain of haptoglobin from patients as potential biomarkers for acute MI and predictors of cardiac remodeling. [28,29] Proteomic analyses using left ventricular tissue from post-MI animals have identified the substrates for matrix metalloproteinases (MMP7 and MMP9). [30,31] This study, however, is the first to specifically identify small molecular weight peptides that are related to post-MI development of HF and therapeutic response of ACE inhibition with Ram in this animal model.

The major difficulty in mining low abundance biomarkers from plasma or serum is the presence of a small number of proteins including albumin, α2-macroglobulin, transferrin and immunoglobulin, that represent as much as 80% of the total protein. The high abundance of these proteins makes it difficult to identify lower abundance protein in plasma using traditional proteomic techniques. Much of the literature on plasma biomarker identification relates to relatively high abundance, high mass proteins identified with conventional proteomic techniques. The existing opportunities to identify signature changes in subsets of lower abundance peptides that may be informative of disease stage remain relatively under-explored. Hence, we have developed a mass spectrometry-based peptide profiling strategy to identify and characterize changes in small molecular weight peptides from plasma samples. The strength of this approach is that it affords opportunity to identify (at the same time) differentially expressed peptide peaks. This provides considerable advantage over conventional approaches where most often the large high abundance proteins are identified.

## Conclusions

The results from this study may potentially be useful in prognosis, diagnosis, and monitoring the treatment response with experimental animal models of MI and HF. The data obtained may be utilized in three ways (i) as components within a multivariate classification model that may be used as a prognostic or diagnostic test; as the identity of the contributing peptides can be determined (ii) specific multiplex immunoassays developed to provide quantitative endpoints and potentially monitor changes with therapeutic intervention; (iii) to increase our understanding of the etiology of cardiovascular disease complications.

## Competing interests

The authors declare that they have no competing interests.

## Authors’ contributions

BW carried out the study design, data collection and analysis, and draft of manuscript. SR carried out proteomic analysis and data collection. MB carried out mass spectrometry analysis. AK carried out the in vivo animal study and sample preparation. MA carried out proteomic analysis and data collection. HK carried out study design and help draft the manuscript. GR carried study design, data analysis and help the draft manuscript. All authors read and approved the final manuscript.
